# Distribution of SARS-CoV-2 Variants in a Large Integrated Health Care System — California, March–July 2021

**DOI:** 10.15585/mmwr.mm7040a4

**Published:** 2021-10-08

**Authors:** Deborah E. Malden, Katia J. Bruxvoort, Hung Fu Tseng, Bradley Ackerson, Soon Kyu Choi, Ana Florea, Julia Tubert, Harpreet Takhar, Michael Aragones, Vennis Hong, Carla A. Talarico, John M. McLaughlin, Lei Qian, Sara Y. Tartof

**Affiliations:** ^1^Department of Research and Evaluation, Kaiser Permanente Southern California, Pasadena, California; ^2^Epidemic Intelligence Service, CDC; ^3^Department of Epidemiology, School of Public Health, University of Alabama at Birmingham, Birmingham, Alabama; ^4^Department of Health System Science, Kaiser Permanente Bernard J. Tyson School of Medicine, Pasadena, California; ^5^Moderna, Inc., Cambridge, Massachusetts; ^6^Pfizer Inc., Collegeville, Pennsylvania.

Data from observational studies demonstrate that variants of SARS-CoV-2, the virus that causes COVID-19, have evolved rapidly across many countries (*1*,*2*). The SARS-CoV-2 B.1.617.2 (Delta) variant of concern is more transmissible than previously identified variants,* and as of September 2021, is the predominant variant in the United States.^†^ Studies characterizing the distribution and severity of illness caused by SARS-CoV-2 variants, particularly the Delta variant, are limited in the United States (*3*), and are subject to limitations related to study setting, specimen collection, study population, or study period (*4*–*7*). This study used whole genome sequencing (WGS) data on SARS-CoV-2–positive specimens collected across Kaiser Permanente Southern California (KPSC), a large integrated health care system, to describe the distribution and risk of hospitalization associated with SARS-CoV-2 variants during March 4–July 21, 2021, by patient vaccination status. Among 13,039 SARS-CoV-2–positive specimens identified from KPSC patients during this period, 6,798 (52%) were sequenced and included in this report. Of these, 5,994 (88%) were collected from unvaccinated persons, 648 (10%) from fully vaccinated persons, and 156 (2%) from partially vaccinated persons. Among all sequenced specimens, the weekly percentage of B.1.1.7 (Alpha) variant infections increased from 20% to 67% during March 4–May 19, 2021. During April 15–July 21, 2021, the weekly percentage of Delta variant infections increased from 0% to 95%. During March 4–July 21, 2021, the weekly percentage of variants was similar among fully vaccinated and unvaccinated persons, but the Delta variant was more commonly identified among vaccinated persons then unvaccinated persons overall, relative to other variants. The Delta variant was more prevalent among younger persons, with the highest percentage (55%) identified among persons aged 18–44 years. Infections attributed to the Delta variant were also more commonly identified among non-Hispanic Black persons, relative to other variants. These findings reinforce the importance of continued monitoring of SARS-CoV-2 variants and implementing multiple COVID-19 prevention strategies, particularly during the current period in which Delta is the predominant variant circulating in the United States.

KPSC facilities represent 15 large medical centers that provide care to approximately 4.6 million members across Southern California. As of 2021, KPSC performs molecular SARS-CoV-2 testing for all patients upon request, regardless of symptoms, and before hospital admission or medical procedures. During March 4–July 21, 2021, specimens were primarily collected via nasopharyngeal or oropharyngeal swab, but self-collection of saliva was also available. During this period, WGS was performed in accredited laboratories on all specimens collected by KPSC facilities that yielded a positive SARS-CoV-2 test result.^§^^,^^¶^ The four most commonly identified SARS-CoV-2 variants were defined according to the CDC classification system as of September 2021.** All other identified variants were grouped together as ‘other’ variants. WGS data were linked with patient electronic medical records. The distributions (frequency and percentage) of variants were compared by week of specimen collection, vaccination status,^††^ age, sex, race/ethnicity, and underlying medical conditions^§§^ (*8*). Differences between groups were calculated using chi-square tests; statistical significance was defined as p<0.05. 

Patients were followed up for 14 days from the date of SARS-CoV-2 specimen collection. COVID-19–related hospitalization was defined as hospital admission from 2 days before until 14 days after the SARS-CoV-2 positive test result. For patients hospitalized 0–2 days before the date of specimen collection, medical chart reviews were conducted to confirm that the hospitalization was related to COVID-19.^¶¶^ For patients hospitalized during the 14 days after the specimen collection date, it was assumed that the hospitalization was related to the COVID-19 diagnosis. Cox proportional hazards regression analysis was used to obtain the adjusted hazard ratio (aHR) and corresponding 95% confidence interval (CI) for the risk for COVID-19 hospitalization associated with the Delta variant (i.e., the predominant variant) relative to all other variants. Regression models were stratified by vaccination status, and were adjusted for age, sex, race/ethnicity, presence of underlying medical conditions, and study period. Data analyses were performed using SAS (version 9.4; SAS Institute). This activity was reviewed by CDC and was conducted consistent with applicable federal law and CDC policy.***

Among 6,798 sequenced SARS-CoV-2–positive specimens collected from KPSC patients during March 4–July 21, 2021, a total of 5,994 (88%) were collected from unvaccinated persons, 648 (10%) from fully vaccinated persons, and 156 (2%) from partially vaccinated persons (Table) (Supplementary Figure 1, https://stacks.cdc.gov/view/cdc/110072). Approximately 45% of all positive SARS-CoV-2 specimens during March 4–July 21, 2021, failed sequencing; specimens most likely to fail sequencing were those collected from vaccinated persons, non-Hispanic Asians persons, older persons (those aged ≥65 years), and those with underlying medical conditions. Compared with unvaccinated persons, fully vaccinated persons were older and included a larger proportion of non-Hispanic Asian persons and persons with multiple underlying conditions. During March 4–May 19, 2021, the weekly percentage of infections attributed to the Alpha variant increased steadily from 20% to approximately 67%, after which it declined. During April 15–July 21, 2021, the weekly percentage attributed to the Delta variant increased from 0% to 95% of all sequenced specimens (Figure).

**TABLE:** Characteristics of study participants with sequenced SARS-CoV-2 specimens, by variant — California, March–July 2021

Characteristic	Variant, no. (%)				p-value^§^
	All positive specimens	Alpha (B.1.1.7)	Gamma (P.1, P.1.1 and P.1.2)	Delta (B.1.617.2, AY.1, AY.2 and AY.3)	
**Total**	**6,798 (100)**	**2,176 (100)**	**509 (100)**	**2,156 (100)**	**N/A**
Vaccination status*					<0.001
Fully	648 (9.5)	84 (3.9)	39 (7.7)	469 (21.8)	
Partially	156 (2.3)	48 (2.2)	20 (3.9)	33 (1.5)	
Unvaccinated	5,994 (88.2)	2,044 (93.9)	450 (88.4)	1,654 (76.7)	
**Sex**					0.415
Female	3,640 (53.6)	1,160 (53.3)	289 (56.8)	1,167 (54.1)	
Male	3,157 (46.4)	1,016 (46.7)	220 (43.2)	989 (45.9)	
Other	1 (—)	0 (—)	0 (—)	0 (—)	
**Age group, yrs**					<0.001
<12	585 (8.6)	200 (9.2)	35 (6.9)	193 (9.0)	
12–17	524 (7.7)	181 (8.3)	34 (6.7)	153 (7.1)	
18–44	3,469 (51.0)	1,060 (48.7)	264 (51.9)	1,192 (55.3)	
45–64	1,823 (26.8)	620 (28.5)	124 (24.4)	495 (23.0)	
65–74	291 (4.3)	83 (3.8)	38 (7.5)	87 (4.0)	
≥75	106 (1.6)	32 (1.5)	14 (2.8)	36 (1.7)	
**Median (IQR)**	35 (23–50)	36 (23–50)	37 (26–52)	33 (23–47)	
**Race/Ethnicity**					<0.001
Hispanic	2,988 (44.0)	909 (41.8)	222 (43.6)	850 (39.4)	
Asian, non-Hispanic	337 (5.0)	83 (3.8)	13 (2.6)	143 (6.6)	
Black, non-Hispanic	822 (12.1)	254 (11.7)	78 (15.3)	353 (16.4)	
White, non-Hispanic	2,045 (30.1)	724 (33.3)	154 (30.3)	619 (28.7)	
Other/Unknown	606 (8.9)	206 (9.5)	42 (8.3)	191 (8.9)	
**Charlson Comorbidity Index^†^**					0.586
0	5,510 (81.1)	1,739 (79.9)	403 (79.2)	1,773 (82.2)	
1	791 (11.6)	275 (12.6)	62 (12.2)	237 (11.0)	
2	250 (3.7)	84 (3.9)	20 (3.9)	76 (3.5)	
≥3	247 (3.6)	78 (3.6)	24 (4.7)	70 (3.3)	

**Abbreviations:** FDA = Food and Drug Administration; IQR = interquartile range; N/A = not applicable.

* Vaccination status was defined as follows: fully vaccinated persons had completed all recommended doses of an FDA–authorized COVID-19 vaccine, including Pfizer-BioNTech, Moderna, and Janssen (Johnson & Johnson) ≥14 days before the positive SARS-CoV-2 test date; partially vaccinated persons had completed a single dose of Pfizer-BioNTech or Moderna COVID-19 vaccine ≥14 days before the positive SARS-CoV-2 test date; unvaccinated persons had no record of receiving a FDA-authorized COVID-19 vaccine ≥14 days before the positive SARS-CoV-2 test date.

^†^ Charlson Comorbidity Index is a weighted composite score based on 17 comorbidities.

^§^ Chi-square test for heterogeneity.

**FIGURE:**
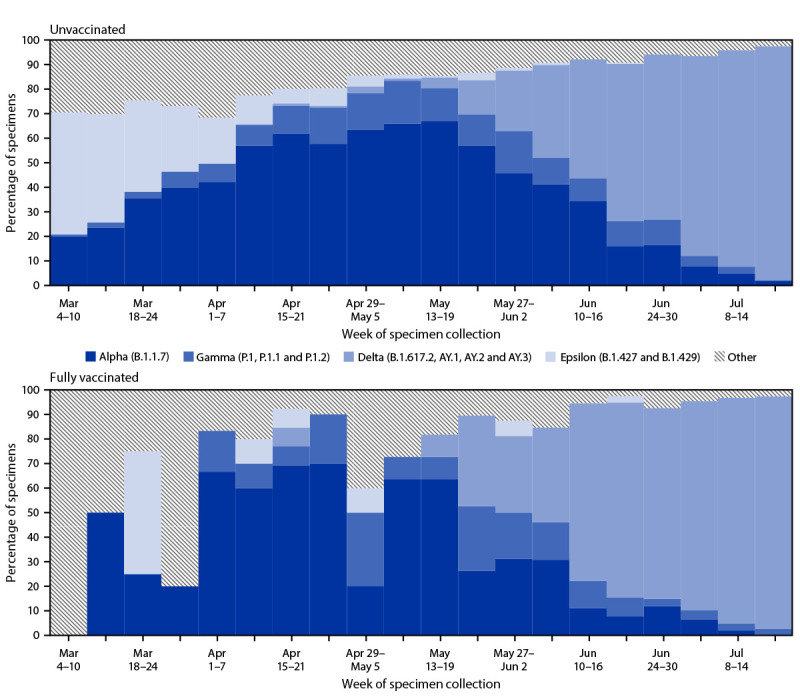
Percentage of SARS-CoV-2 variants* identified among sequenced specimens, by unvaccinated (n = 5,994) and fully vaccinated (n = 648) status^†^ — California, March–July 2021 **Abbreviation: **FDA = Food and Drug Administration. * Variants and their associated SARS-CoV-2 (Pango) lineages were defined according to the CDC classification system at the time of the report (https://www.cdc.gov/coronavirus/2019-ncov/variants/variant-info.html). The four most commonly identified variants were displayed separately, and all other identified lineages were grouped together as other variants. ^^†^^ Fully vaccinated persons had completed all recommended doses of an FDA-authorized COVID-19 vaccine, including Pfizer-BioNTech, Moderna, and Janssen (Johnson & Johnson) ≥14 days before the positive SARS-CoV-2 test date. Unvaccinated persons had no record of receiving an FDA-authorized COVID-19 vaccine ≥14 days before the positive SARS-CoV-2 test date. Partially vaccinated persons had completed a single dose of Pfizer-BioNTech or Moderna COVID-19 vaccine ≥14 days before the positive SARS-CoV-2 test date; these persons were not included in the current analysis because of sample size limitations.

The absolute number of specimens that yielded a positive SARS-CoV-2 result was much lower among fully vaccinated persons (648) than among unvaccinated persons (5,994). In general, the weekly percentages of SARS-CoV-2 variants among fully vaccinated persons approximately mirrored those among unvaccinated persons (Supplementary Figure 2, https://stacks.cdc.gov/view/cdc/110120). However, overall, the percentage of fully vaccinated persons with infections attributed to the Delta variant was slightly higher (22%) than the percentage infected with other variants (4%–8%). There were slight differences in the distribution of selected variants by age group and race/ethnicity, but the distribution did not substantially differ between males and females, or between patients with and without multiple underlying medical conditions (Table). Compared with all infections, those from the Delta variant were slightly more common among non-Hispanic Black persons (16.4% versus 12.1%, respectively). Infections attributed to the Delta variant were also more common among younger persons, with the majority of infections identified among persons aged 18–44 years (55.3%). Twenty-five (3.9%) fully vaccinated patients and 393 (6.6%) unvaccinated patients were admitted to hospital within 2 days before to 14 days after the specimen collection date. Among unvaccinated persons, infection with the Delta variant compared with all other variants was associated with an increased adjusted risk of hospitalization (aHR = 1.81, 95% CI = 1.30–2.52).

## Discussion

In this study, conducted within a large integrated health care system in southern California, the weekly percentage of all infections attributed to the Delta variant rapidly increased to 95% during March 4–July 21, 2021. Infection with the Delta variant was more common among younger persons (aged 18–44 years) and among non-Hispanic Black persons. The Delta variant was associated with an apparent increased risk of hospitalization among unvaccinated persons. These findings reinforce the importance of implementing multicomponent COVID-19 prevention strategies, particularly vaccination among eligible populations.

Consistent with national and global sequencing data, a rapid change in the distribution of SARS-CoV-2 variants was observed, with Alpha becoming the dominant variant between approximately mid-April and late-May 2021, and Delta quickly becoming the dominant variant thereafter (*3**,**4*). Similar to previous reports, persons with infections attributed to the Delta variant were younger, relative to all persons with positive sequenced specimens. This could be due to multiple factors, including increasing vaccination coverage among older adults and increased social interactions among younger adults during periods when the Delta variant predominated (*3*,*4*). Similarly, the observed differences in prevalence of Delta variant infections across race/ethnicity categories might reflect differences in risk for COVID-19 exposure among these persons during periods of high Delta variant transmission.

In general, the weekly percentages of isolated variants in this population of KPSC members were similar by vaccination status, but cumulatively, from March 4 to July 21, 2021, the total percentage of infections attributed to the Delta variant was higher among fully vaccinated persons than among unvaccinated persons, relative to other variants. Previous studies have attributed this to either a possible reduction in vaccine efficacy associated with Delta (*3*,*4*) or to the coincidental waning of vaccine-induced immunity in certain subpopulations (e.g., those vaccinated earlier in the pandemic) during recent periods when Delta variant transmission was high.^†††^ Compared with other variants, infections attributed to Delta were associated with an observed increased risk of hospitalization among unvaccinated persons, aligning with previous reports that infection with the Delta variant appears to result in more severe disease (*3*,*4*,*9*). However, this finding could also be the result of systematic differences in the testing behavior or clinical risk factors of persons with infections attributed to the Delta variant relative to other variants.

The findings in this report are subject to at least five limitations. First, approximately 45% of specimens were not successfully sequenced, and therefore, the study population was not representative of all positive specimens in this population. Sequence success rates are correlated with the amount of viral genetic material in the specimen, which can be influenced by factors such as age, vaccination status, variant, or type of specimen, as observed in the current report. Second, community testing was largely self-selected; therefore, testing patterns might have differed between vaccinated and unvaccinated patients, or among patients infected with different variants. However, models were adjusted for study period to control for potential changing testing behaviors throughout the pandemic. Third, patients with infections attributed to different variants might have differed systematically in other respects not covered in the current report, which in turn might have affected COVID-19 severity. Fourth, the numbers of partially vaccinated and fully vaccinated persons were small, limiting the power for these subgroup analyses and precluding comparisons of hospitalization among vaccinated persons. Finally, KPSC patients were possibly tested elsewhere; specimens from these patients would not be included in the current data.

These findings reinforce the importance of continued monitoring of SARS-CoV-2 variants and implementing multicomponent COVID-19 prevention strategies, particularly during the current period in which Delta is the predominant circulating variant in the United States. Such preventive strategies include increasing COVID-19 vaccination coverage among eligible populations in coordination with other strategies such as universal masking and physical distancing.

**Summary**

**What is already known about this topic?**

The highly transmissible SARS-CoV-2 (B.1.617.2) Delta variant is the predominant variant circulating in the United States.

**What is added by this report?**

During March 4–July 21, 2021, sequencing data from 6,798 SARS-CoV-2–positive specimens were linked to electronic health records among Kaiser Permanente Southern California members. The weekly percentage of all infections attributed to the Delta variant rapidly increased to 95% during this period. Infection with the Delta variant was more common among younger persons and among non-Hispanic Black persons.

**What are the implications for public health practice?**

These findings reinforce the importance of continued monitoring of SARS-CoV-2 variants and implementing multicomponent COVID-19 prevention strategies, particularly during the current period in which Delta is the predominant circulating variant in the United States.

## Footnotes

*
https://www.medrxiv.org/content/10.1101/2021.06.03.21258293v1.full.pdf

† https://covid.cdc.gov/covid-data-tracker/#variant-proportions (Accessed September 27, 2021).

§ Specimens were tested at KPSC laboratories with FDA-authorized real-time reverse transcription–polymerase chain reaction (RT-PCR) using the TaqPath COVID-19 High-Throughput Combo Kit on the Thermo Fisher Scientific Amplitude Solution (Thermo Fisher Scientific, Waltham, Massachusetts) or using the cobas SARS-CoV-2 assay on the cobas 8800 System or the cobas SARS-CoV-2 & Influenza A/B Assay on the cobas Liat Analyzer (Roche Diagnostics, Indianapolis, Indiana). Specimens were sequenced using the NovaSeq 6000 Sequencing System S1 flow cell, which included the NovaSeq 6000 S1 Reagent Kit v1.5 (300 cycles) and the NovaSeq 6000 Sequencing System (Illumina Inc., San Diego, California).

¶ Specimens sequenced before March 4, 2021 were excluded from the analyses because their collection preceded the mass sequencing project; therefore, these specimens were not representative of all positive specimens and were not sequenced using the standardized protocol outlined in the current report.

** https://www.cdc.gov/coronavirus/2019-ncov/variants/variant-info.html (Accessed September 27, 2021).

†† Vaccination status was defined as follows: Fully vaccinated persons had completed all recommended doses of an FDA–authorized COVID-19 vaccine, including Pfizer-BioNTech, Moderna, and Janssen (Johnson & Johnson) ≥14 days before the positive SARS-CoV-2 test date; unvaccinated persons had no record of receiving an FDA–authorized COVID-19 vaccine ≥14 days before the positive SARS-CoV-2 test date. Partially vaccinated persons had completed a single dose of Pfizer-BioNTech or Moderna COVID-19 vaccine ≥14 days before the positive SARS-CoV-2 test date.

§§ Underlying medical conditions were defined according to the weighted Charlson Comorbidity Index, which included conditions of interest available in electronic medical records from the 12 months before the date of SARS-CoV-2 test. Conditions included in the Charlson index were immunosuppressive disorders, acute myocardial infarction, congestive heart failure, peripheral vascular disease, cerebrovascular disease, dementia, chronic obstructive pulmonary disease, rheumatoid disease, peptic ulcer disease, mild and moderate/severe liver disease.

¶¶ Medical records of all patients with a hospital admission on the same day or 1–2 days before their positive SARS-CoV-2 specimen collection date were reviewed for possible COVID-19–related codes or medical notes. Patients were considered to have a confirmed COVID-19 related hospital admission if their medical records indicated that COVID-19 was either the primary reason for the admission or for extension of hospitalizations that were initially for an unrelated condition.

*** 45 C.F.R. part 46, 21 C.F.R. part 56; 42 U.S.C. Sect. 241(d); 5 U.S.C. Sect. 552a; 44 U.S.C. Sect. 3501 et seq.

†††
https://papers.ssrn.com/sol3/papers.cfm?abstract_id=3909743
